# Optimization of Ultrasound-Assisted Extraction of Phloretin and Other Phenolic Compounds from Apple Tree Leaves (*Malus domestica* Borkh.) and Comparison of Different Cultivars from Estonia

**DOI:** 10.3390/antiox10020189

**Published:** 2021-01-28

**Authors:** Sana Ben-Othman, Hedi Kaldmäe, Reelika Rätsep, Uko Bleive, Alar Aluvee, Toonika Rinken

**Affiliations:** 1 ERA Chair for Food (By-) Products Valorisation Technologies VALORTECH, Estonian University of Life Sciences, Kreutzwaldi 56/5, 51006 Tartu, Estonia; reelika.ratsep@emu.ee; 2 Polli Horticultural Research Centre, Estonian University of Life Sciences, Uus 2, 69108 Polli, Estonia; hedi.kaldmaee@emu.ee (H.K.); uko.bleive@emu.ee (U.B.); Alar.Aluvee@emu.ee (A.A.)

**Keywords:** *Malus domestica*, leaves, agri-food waste valorisation, polyphenols, phloretin, antioxidant activity, ultrasound-assisted extraction, optimization

## Abstract

Polyphenolic compounds, plant secondary metabolites essential for plant survival, are known for their high antioxidant and anti-inflammatory activity. In addition, several polyphenols, such as phloretin, also have potential antiviral effects, making these compounds potential ingredients of biofunctional foods. A promising source for the extraction of phloretin is a by-product of apple production—apple tree leaves. Focusing on green technologies, the first aim of the present study was to optimize the direct ultrasound-assisted extraction conditions to gain the maximum yield of phloretin from air-dried apple leaves. For the optimization of process parameters, we applied the response surface method with Box–Behnken design. The optimal extraction conditions were extraction time 14.4 min, sonication amplitude 10% and 10 g of sample per 100 mL solvent (70% ethanol, *w*/*w*). Using these conditions, we assessed the content of individual and total polyphenolic compounds along with antioxidant activity in the leaves of different autumn and winter apple cultivars grown in Estonia. The analyses were carried out with chromatographic (HPLC-DAD-MS/MS) and spectrophotometric methods. The phloretin concentration ranged from 292 to 726 µg/g and antioxidant activity from 6.06 to 11.42 mg GA eq./g, these being the highest in the local winter cultivars ‘Paide taliõun’ and ‘Tellissaare’, respectively.

## 1. Introduction

There are over 8000 polyphenolic compounds found in plant-derived material [1]. The polyphenolic compounds are known for their high antioxidant and anti-inflammatory activity, which favorably affects health risks for metabolic disorders [2]. Several polyphenols of plant origin, such as phloretin, quercetin, etc., are reported to have remarkable antiviral effects, preventing the attachment and replication of viruses and stimulating immune responses [3]. The presence of phenolic compounds in the diet has also been related to positive effects on cognitive decline, asthma and pulmonary function, bone health, and weight control [4]. In herbal therapy, natural polyphenols have been used in the form of infusions and extracts for treating ailments of digestive, vascular, urinary, and respiratory systems, in dermatology and for anticancer therapy [5]. Their main role is considered to consist in counteracting oxidative stress at the cellular level [6]. The health effects of these compounds are dependent on their amount in daily intake and their bio-availability [2].

The effects of polyphenols on human health make these compounds prospective components in the production of functional foods [7]. Besides the enrichment of food products, these compounds are also used in practical healthcare for the production of dietary supplements and cosmetic preparations [8], e.g., phloretin as a natural skin whitening agent due to its ability to inhibit tyrosinase activity [9]. In experimental cell models, the antioxidant effect of phloretin towards different stressors has been reported already at concentrations of 0.27 μg/mL [10].

Apple trees (*Malus domestica* Borkh.) are grown widely in different climate zones throughout the world, thus placing apples among the major fruits on the market [11], but they are also a potential source of antioxidant polyphenolic compounds. The most abundant phenolic compounds in apples are chlorogenic acid, phloretin, phloridzin, epicatechin, quercetin, and procyanidin B2 [12,13]. Levels of total phenolics and individual phenolic compounds with diverse antioxidant properties vary in different parts of apple trees (and fruits), but also between apple cultivars [13,14]. It has been established that various polyphenols, and in particular phloridzin and phloretin, are most abundant in apple leaves [12,15] and twigs [16], while procyanidins predominate in apple fruits [12,15]. The polyphenolic concentration in fruits peaks early in the season, and decreases during fruit development [1]. Large amounts of apple tree leaves, as well as unripe fruits, are harvested during summer pruning season to improve the apple production quality. This generates large-scale by-products that are a valuable source of polyphenolic compounds with high antioxidant potential [1,12,17]. 

Phenolic compounds are plant secondary metabolites required for plant survival [18]. Following the attacks of pathogenic bacteria and fungi, changes in the content of phenolics, particularly phloridzin and phloretin, have been detected both in resistant and susceptible apple cultivars [1,19]. It is also interesting to note that after inoculation of *Erwinia amylovora*, the phloretin content increased at a higher rate in resistant cultivars, although the initial concentrations of phloridzin and phloretin in the leaves were similar [1]. 

Dihydrochalcone phloretin and its glucoside phloridzin are found to be the major phenolic components in apple leaves. Depending on the cultivar and leaf maturation stage (terminal, medium or basal stage), phloridzin represented from 5.4% to 14% of leaf dry weight [15]. Greater variations were found for phloretin concentrations among different cultivars and leaf ages when compared to phloridzin [15]. In young apple leaves and twigs, phloridzin can account for up to 10% of dry weight [16]. Phloretin and phloridzin have been also found in apple seeds [20]. For the industrial production of dihydrochalcones, root bark of the apple tree is commonly used as a raw material. However, apple leaves contain similar concentrations of dihydrochalcones and have the advantage of being more abundant and renewable compared to the root bark, which can be harvested once in the tree’s life cycle [12,17].

Phloridzin plays an important role as a dietary polyphenol, with its capacity to reduce intestinal glucose uptake. In a recent study, Niederberger et al. [21] reported that the estimated average dietary phloridzin intake in Europe was low compared to levels used in human studies, in which phloridzin had a positive effect on glucose uptake. The author suggested that an increased dietary intake of phloridzin could have positive effects on the development and progression of some diet-related chronic diseases. Thus, apple leaves extracts have an interesting potential use for the enrichment of food products with phloridzin.

Today, studies focusing on the recovery of polyphenol extracts from apple leaves and information about the optimal extraction methods and conditions are still scarce. In Table 1, we have summarized different solvents and experimental procedures used for the extraction of dihydrochalcones from apple tree leaves and wood, available from recent literature reports. The collected data indicate that the yields of these compounds recovered through extraction are significantly dependent on the extraction procedure, but also vary between different cultivars. 

A well-recognized green extraction technique allowing the full extraction of bioactive compounds in a short time with high reproducibility, reduced solvent consumption, and lower energy consumption is ultrasound-assisted extraction (UAE) [27]. The optimization of UAE allows one to increase the extraction yield while preserving the extract’s biological activity, and to prevent the wastage of raw material and solvent [28]. Indirect sonication in ultrasound (US) water baths, wherein the extraction medium is not in direct contact with the US source, has been used in earlier studies (Table 1) [8,12,22]. However, direct contact between the US source and the extraction medium allows one to intensify the cavitation effect and improve the extraction yield of bioactive compounds from the plant cell matrix [28].

The objective of the present study was to investigate the potential of apple tree leaves of different Estonian autumn and winter apple cultivars as an under-utilized source for the recovery of polyphenolic compounds. To gain the maximal phloretin yield and retain the maximum antioxidant activity of polyphenolic compounds during extraction, we used a US probe, exposing samples to direct sonication for the extraction of the targeted compounds from air-dried leaves. For the extraction, we used environmental friendly ethanol:water solutions. First, we optimized the UAE parameters, such as sonication amplitude, extraction time, and sample to solvent ratio, by applying the response surface methodology (RSM). After determination of the optimum extraction conditions, we compared extracts from different local apple cultivars in terms of antioxidant activity, total phenolic content and concentration of different individual phenolic compounds.

## 2. Materials and Methods 

### 2.1. Selection of Apple Tree Cultivars

Two different autumn apple cultivars ‘Tiina’ and ‘Liivi kuldrenett’, and five winter apple cultivars ‘Tellissaare’, ‘Karksi renett’, ‘Paide taliõun’, ‘Talvenauding’ (all bred in Estonia), and “Cortland” (bred in US) were chosen for the study. These cultivars are all appreciated for the taste of fruits, and are widely grown in the region. Most of them are also recommended for commercial production orchards. In addition, all these cultivars have medium or high susceptibility to apple scab [29]. In the case of ‘Tiina’ and ‘Liivi kuldrenett’, the leaves are more susceptible to apple scab than the fruit. 

### 2.2. Collection of Apple Tree Leaves and Preparation of Samples

The apple tree lateral shoots with leaves were collected from a private orchard in Tartu County, South Estonia (58°23′ N, 26°84′ E), during the summer pruning season in July 2020. The material was air-dried, and the leaves were removed from the shoots and stored at room temperature. The dried samples were ground to a fine powder using a cutting mill SM 300 (Retsch, Haan, Germany) at 1500 rpm with a 2 mm bottom sieve, reaching a final particle size of <1 mm.

### 2.3. Extraction Procedure

The ultrasound-assisted extraction of polyphenolic compounds from dried apple tree leaves was performed using a Digital Sonifier^®^ S450 CE equipped with a 13 mm diameter disruptor horn (400 W Power, 20 kHz Frequency; Branson Ultrasonics Co., Danburry, CT, USA). Dried leaf powder (1–10 g) was mixed with 100 mL ethanol–water solution (70:30, *w*/*w*) in a double-wall glass tempering beaker connected to a circulating tap water to avoid heating during the extraction. The obtained extract was separated from the residual plant material by vacuum filtration through paper filter (12–15 µm retention rate, grade 1288; Sartorius AG, Göttingen, Germany). Recovered extracts were then analyzed for individual phenolic compounds and total phenolic content, and DPPH free radical scavenging activity.

### 2.4. RSM Design for the Optimization of Extraction

For the analysis of the influence of three major input variables of the UAE process on the extraction efficiency of total phenolic content (TPC), antioxidant activity, and selected phenolic compounds (incl. phloretin, phloridzin quercetin and others), we used the response surface methodology (RSM) coupled with a Box–Behnken design. The selected variables for optimization were extraction time (A, min), sonicator amplitude (B, %), and sample to solvent ratio (C, g sample/100 mL solvent). The variation ranges of input factors were 5–30 min, 10–100% and 1–10 g/100 mL solvent, and these were coded for the design and analyses. The complete design included 17 runs with 5 runs for the central point (Table 2).

The experimental design and analyses of the results were carried out with the Design-Expert^®^ software (ver.12, Stat-Ease Inc., Minneapolis, MN, USA).

### 2.5. Determination of Total Phenolic Content

Total polyphenols content (TPC) was measured using the modified Folin–Ciocalteau (FC) method [30]. In brief, the gallic acid (GA) standards were prepared with the following concentrations: 50, 150, 250, 350, and 400 µg/mL. For the calibration, 0.4 mL of each standard was injected into a 4 mL spectrophotometer cuvette, to which 2.0 mL of FC reagent (0.2 N) was added, and after 5 min 1.6 mL of Na_2_CO_3_ (75 g/L) was added and the samples were incubated for 60 min in the dark at room temperature. Prior to the analyses of apple leaf extracts, the crude samples were diluted 40 times (250 µL of crude sample + 9750 µL of 70% *w*/*w* aqueous ethanol). The measurement procedure was the same as for the standard calibration described previously. The absorbance values of the samples were measured at 760 nm using a spectrophotometer (UV-1800, Shimadzu, Kyoto, Japan). The results were expressed in mg of gallic acid equivalents per g of dry weight (mg GA eq./g dw). All chemicals used were of laboratory grade and purchased from Sigma (Steinheim, Germany).

### 2.6. DPPH Free Radical Scavenging Activity

Free radical scavenging activity measurements were performed in duplicate using a 2.2-diphenyl-picrylhydrazyl (DPPH) assay with slight modifications [31]. Briefly, the gallic acid calibration for the analysis was prepared as follows: 0.125, 0.100, 0.0625, 0.050, 0.025 and 0.010 mg/mL. For the measurement, 0.1 mL of each standard was pipetted into a 4 mL spectrophotometer cuvette, to which 3.7 mL of DPPH radical (63.5 µM) was added. The samples were incubated for 60 min in the dark at room temperature. The analytical procedure of the previously diluted apple leaf extracts was the same as for the standard calibration described previously. The absorbance values of the samples were measured at 515 nm using a spectrophotometer (UV-1800, Shimadzu, Japan). The results were expressed in mg of gallic acid equivalent per g of dry weight (mg GA eq./g dw). All chemicals used were of laboratory grade and purchased from Sigma (Steinheim, Germany).

### 2.7. Identification and Quantification of Polyphenols by LC-MS Method

Qualitative and quantitative analyses were performed on a Shimadzu Nexera X2 UHPLC with mass spectrometer LCMS 8040 (Shimadzu Scientific Instruments, Kyoto, Japan). The UHPLC system was equipped with a binary solvent delivery pump LC-30AD, an autosampler Sil-30AC, column oven CTO-20AC and diode array detector SPD-M20A. A reverse phase column ACE Excel 3 (C18, PFP, 100 × 2.1 mm; from ACE^®^ Advanced Chromatography Technologies Ltd., Aberdeen, Scotland) and pre-column (SecurityGuard ULTRA, C18; from Phenomenex, Torrance, CA, USA) were used at 40 °C for the separation of individual polyphenols. The flow rate of the mobile phase was 0.25 mL/min, and the injected sample size was 1 µL or 0.2 µL depending on the concentration of the sample. Mobile phases consisted of 1% formic acid in Milli-Q water (mobile phase A) and 1% formic acid in methanol (mobile phase B). Separation was carried out for 40 min under the following conditions: gradient 0–27 min, 15–80% B; 27–29 min, 80–90% B; 29–35 min, isocratic 90% B, and re-equilibration of the system with 15% B 8 min prior to the next injection. All samples were kept at 4 °C during the analysis. 

The total polyphenol content, expressed as mg chlorogenic acid equivalent per g of dry weight (mg ChlA eq./g dw), and total flavonols expressed as mg quercetin equivalent per g of dry weight (mg Q eq./g dw) were quantified at the wavelengths of 280 nm and 360 nm, respectively [32].

Individual phenolic compounds were identified by comparing the retention times, UV spectra, and parent and daughter ion masses with those of the standard compounds. MS data acquisitions were performed on LCMS 8040 with the ESI source operating in both positive and negative modes. The interface voltage was set to 4.5 kV (both ESI+ and ESI−). Nitrogen was used as the nebulizing gas (3 L/min) and drying gas (15 L/min). The heat block temperature was 350 °C and the desolvation line (DL) temperature was 250 °C. All samples were analyzed in triplicate, and the results were expressed as mg per g of dry weight (mg/g dw). Retention times and mass spectral data of standard phenolic compounds are summarized in Table 3.

All standards (chlorogenic acid, *p*-coumaric acid, caffeic acid, epicatechin, phloridzin dihydrate, phloretin, quercetin-3-d-galactoside, quercetin-3-d-glucoside, kaempferol-3-glucoside, quercitrin hydrate, rutin) and chemicals (formic acid, methanol) used were of analytical grade and purchased from Sigma (Steinheim, Germany).

### 2.8. Statistical Analysis

The statistical analysis was conducted using Prism version 5 (GraphPad Software, San Diego, CA, USA). Data were analyzed by one-way analysis of variance (ANOVA), followed by Tukey’s test. The correlation was evaluated by Pearson analysis. Differences at *p* < 0.05 were considered to be significant.

## 3. Results and Discussion

### 3.1. Optimization of Ultrasound-Assisted Extraction of Phloretin

Ultrasound-assisted extraction (UAE) was selected for the extraction of phloretin from apple leaves, as this technique combined with the cooling of the sample allows one to conduct extractions at relatively low temperatures, which is favorable to enhance the extraction yield of heat-sensitive components. There are several independent process parameters which affect UAE, such as extraction time, temperature, solvent composition, power capacity, sample to solvent ratio, issues of sample grinding and mixing, shape of the vessel, and others. Three independent commonly modified factors (extraction time, amplitude of the sonicator, and the sample to solvent ratio) were selected for the optimization of the process. The minimum and maximum levels of these factors for the extraction of phenolic compounds from leaves were established during the preliminary experiments, so we did not expand the design space and used the Box–Behnken design for the optimization of these factors for the UAE process. Considering temperature, we kept this factor constantly below 25 °C with a cooling system to avoid the degradation of the phenolic compounds during the extraction process. Further, as the present study was focused on achieving maximum phloretin yields, requiring maximum interface between the solid and liquid phases as a precondition, we did not optimize the degree of sample grinding and the mixing rate in the extraction vessel, and kept these factors at the maximum possible levels. The choice of 70% ethanol:water solution as an extraction solvent was based on earlier data indicating the highest yields of polyphenols from apple leaves at this particular ethanol:water ratio [8,24,33].

The analysis of experimental results was based on the yield of the targeted individual phenolic compounds, total phenolic content, and the antioxidant activity, with the main focus on maximizing the output of phloretin. Phloretin fulfils the criteria of Lipinski’s rule of five, which would make it a likely orally active drug-like compound in humans, and its bioavailability is 1. The phloretin molecule is lipophilic (log *p* value is 2.2–3.9), and it is practically insoluble in water (0.13 g/L) [34]. 

Based on the summary statistics from model fitting, the best model to maximize the phloretin output was the reduced two-factor interaction (2FI) model (*p* = 0.0116). The 2FI model’s F-value of 5.50 and *p*-value of 0.0116 imply that the model was significant. There was no influence of time-related lurking variables in the background, as the plot of the residuals versus runs showed a random scatter (data not shown). The ANOVA test indicated that the extraction time and its interactions with other studied factors were statistically not significant, so these terms were excluded from the model. The final equation in terms of actual factors was the following:

(1) Y=135.84+2.70×B+29.65×C−0.43×B×C

**Figure 1:**
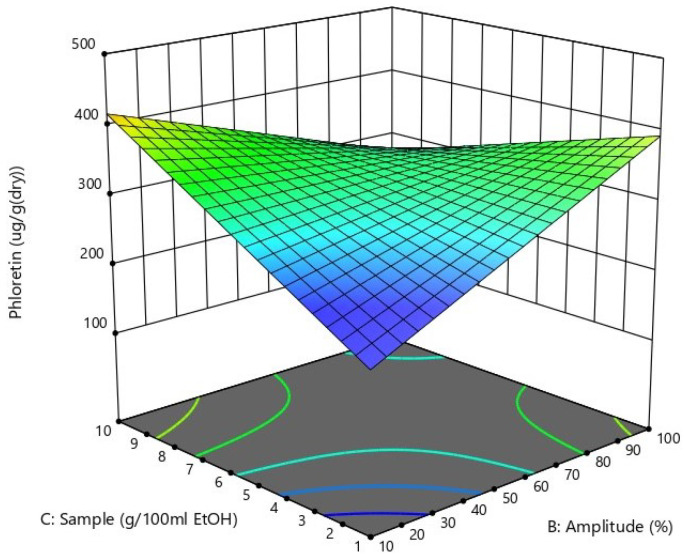
Three-dimensional response surface plot showing the effects of the amount of sample and sonication amplitude on phloretin yield.

The 3D response surface, indicating the effects of sonication amplitude (factor *B*) and sample amount per 100 mL solvent (factor *C*) on phloretin yield, is shown in Figure 1. According to the process model (Equation (1)), the impacts of factors *B* and *C* on phloretin yield are quite similar in actual terms (as the amplitude range is 10 times bigger than the range of sample amount), and the increase in phloretin yield can be achieved both by increasing the sample amount and by decreasing the amplitude, or decreasing the sample amount and increasing the amplitude. High amounts of sample combined with high sonication amplitude led also to a decrease in the yield of phloretin (Figure 1), which is probably caused by the clotting of the sample and the ineffectiveness of stirring in such a thick mixture.

As the phloretin yield was not significantly dependent on extraction time, we also considered the TPC and DPPH values to maximize the efficiency of the extraction of all phenolic compounds. As the phenolic compounds can be degraded at high sonication amplitudes (high energy), the optimal extraction conditions were as follows: extraction time 14.4 min, extraction amplitude 10%, and the amount of sample per 100 mL solvent was 10 g. To assess the effect of sonication on the extraction yield, the optimized protocol was repeated also in silent conditions without sonication. Comparing the yields of polyphenols extracted from the air-dried apple leaves, the extraction yields in silent conditions were from 4 to 7.5 times lower depending on the particular phenolic compound. For phloretin, the sonication increased the yield 6.5-fold. 

### 3.2. Polyphenols Content and Radical Scavenging Activity of Apple Leaves Extracts from Different Cultivars

The TPC, flavonols, and DPPH radical scavenging activity in apple leaf extracts from the seven locally grown cultivars are presented in Table 4. The TPC in the recovered extracts ranged from 35.67 to 57.74 mg GA eq./g dw. When determined by UPLC-DAD analysis, the total polyphenols content was slightly higher, ranging between 37.28 and 71.06 mg ChlA eq./g dw. Nevertheless, both methods showed that the highest polyphenol content was obtained from the leaf powder of cultivar ‘Tellissaare’, followed by cultivars ‘Karksi renett’ > ‘Paide taliõun’ > ‘Cortland’ > ‘Liivi kuldrenett’ > ‘Talvenauding’ > ‘Tiina’. Statistical analysis showed that the TPC in extracts from the ‘Tellissaare’ cultivar was significantly higher than that in all other six cultivars, whereas the TPC was not significantly different between the extracts from cultivars that showed the lowest content (i.e., ‘Liivi kuldrenett’, ‘Talvenauding’, and ‘Tiina’). Previous studies on leaf extracts from different apple cultivars have reported a significant difference in TPC [8,12,22,35]. Parvaneh et al. [35] showed that the rootstock, the cultivar genotype, and their interactions had significant effects on the polyphenol contents of apple leaves, and this in part was related to the activity of enzymes responsible for the biosynthesis of phenolic compounds and flavonoids.

Comparison of the results from previous studies on extracts from apple leaves (Table 1) shows that TPC largely varies according to the preparation method of the raw material, as well as the extraction conditions. The TPC in extracts obtained from lyophilized leaf powder ranged between 56.74 and 163.35 mg GA eq./g dw, while TPC was relatively lower in extracts recovered from dried apple leaf powder, ranging between 24.10 and 37.10 mg GA eq./g dw. In the present study, we used air-dried leaf powder, and, on one hand, the recovered polyphenols content was relatively low compared to extracts recovered from lyophilized leaves. On the other hand, the polyphenol content was higher than in the previously reported results for extracts of air-dried leaves as well as apple tree wood. Lyophilization is widely used for dehydrating and improving the stability of pharmaceutical products. However, due to the high cost of this process, its application is still limited in the food industry [36]. Thus, air-drying seems to be a more suitable treatment for apple leaves prior to extraction, since lyophilization is an expensive process and is more energy-consuming. 

The total flavonols content in apple leaf extracts (Table 4) ranged between 7.47 and 12.23 mg Q eq./g dw, accounting for approximately 16% to 23% of the TPC. Liaudanskas et al. [8] reported a comparable proportion of total flavonoids in the ethanol extracts of apple leaves, ranging between 21% and 27% of the total polyphenol content. Similar to the total polyphenol content, extracts of cultivar ‘Tellissaare’ displayed significantly higher flavonols contents, whereas extracts from the leaves of ‘Paide taliõun’ had the lowest flavonols contents. 

The antioxidant activity of the recovered extracts was determined by evaluating their DPPH free radical scavenging activity, and was expressed as mg GA eq./g dw, as presented in Table 4. Leaf extracts of cultivar ‘Tellissaare’, which showed the highest TPC and flavonols, exhibited the strongest antioxidant capacity, with 48.4% radical scavenging activity equivalent to 11.42 mg GA eq./g dw. The radical scavenging activity of extracts from other cultivars ranged between 40.1% and 28.9% (6.06 and 9.19 mg GA eq./g dw). The correlation between the antioxidant activities of apple leaf extracts and their total polyphenol and flavonols contents was confirmed by Pearson correlation analysis. There was a significant correlation between DPPH radical scavenging activity and the total polyphenols content (*r* = 0.9533, *p* = 0.0009), while the correlation between DPPH radical scavenging activity and flavonols content was not significant (*r* = 0.6758, *p* = 0.0957). A previous study investigating the antioxidant activity of apple leaf extracts reported a strong positive correlation between the total polyphenols and flavonoids contents of the extracts and their antioxidant activities [8]. Teleszko and Wojdyło [22] investigated the antioxidant activity of leaf extracts from different fruit trees and bushes, including apple, quince, chokeberry, cranberry, etc. Their results showed that apple leaf extract exhibited the third-highest content of total polyphenols, whereas it had one of the lowest antioxidant activities, which was explained by the differences in the polyphenols profiles of different plant species.

### 3.3. Identification and Quantification of Individual Phenolic Compounds in Apple Leaves Extracts

Samples were analyzed by UHPLC-MS for the qualitative and quantitative analysis of the individual phenolic compounds present in apple leaf extracts from different cultivars. The method was optimized, and the calibration ranges of standards were adjusted considering the estimated concentrations of polyphenolic compounds in apple leaf extracts. The limit of detection (LOD) and limit of quantification (LOQ) values for the targeted phenolic compounds were far below the actual measured concentrations in the extracts, and ranged from 0.018 to 0.081 μg/mL and 0.060 to 0.271 μg/mL, respectively. The LOD and LOQ values were the highest for kaempferol-3-glucoside and the lowest for quercetin-3-glucoside. For phloretin, the value of LOD was 0.039 μg/mL, and the LOQ was 0.130 μg/mL. 

The distributions of the individual polyphenols in the leaf extracts from apple cultivars under study are presented in Table 5. The results show that apple leaf extracts contained compounds from four polyphenolic groups: phenolic acids, dihydrochalcones, flavonols and catechins. The latter were only present in the form of epicatechin in low concentrations, which were below the detection limit of the quantification method. 

In Section 3.1, the UAE conditions were optimized to maximize the extraction yield of phloretin. Under these optimal extraction conditions, the concentrations of phloretin obtained ranged between 292 and 726 µg/g dw. The phloretin concentration was the highest in the leaves of winter cultivars ‘Paide Taliõun’ and ‘Tellissaare’, with 726 and 505 µg/g, respectively. The concentrations of phloretin recovered in this study were higher than that reported by Rana and Bhushan [24] in extracts from dried apple leaves, which was 150 µg/g. In addition, the recovered phloretin concentrations are comparable to those obtained from apple tree roots (399 µg/g), which are more commonly used as a raw material for the industrial production of phloretin [26]. 

Phloridzin, a phloretin glucoside, was the major phenolic constituent of apple leaf extracts, accounting for 60% to 80% of the total phenolic compounds in different cultivars. The concentrations of phloridzin in the extracts ranged between 11,511 and 31,654 µg/g dw. Phloridzin content was the highest in the leaves of apple cultivar ‘Tellissaare’. These results are in accordance with previous studies reporting phloridzin as the major phenolic constituent of apple leaves [8,12,15,25]. 

Apple leaf extracts also contained flavonols, mainly quercetin glycosides. Quercitrin (quercetin-3-rhamnoside) was the major quercetin glycoside present in the extracts, and concentrations in different cultivars ranged between 1988 and 4290 µg/g dw. The kaempferol-3-glucoside concentrations in different cultivars ranged between 809 and 1680 µg/g dw. Both quercetin and kaempferol aglycones were detected in apple leaf extracts, but their concentrations were very low compared to their glycosides. Their concentrations in different cultivars ranged between 16– and 40 µg/g dw and 2 and 4 µg/g dw, for quercetin and kaempferol, respectively. Chlorogenic acid was the major phenolic acid detected in all studied cultivar extracts, with concentrations ranging between 178 and 338 µg/g dw. Previous studies on apple leaf extracts also reported quercitrin and chlorogenic acid as the major flavonol and phenolic acid in leaf extracts, respectively [8,12].

## 4. Conclusions

The UAE of polyphenolic compounds from apple tree leaves, with the main focus on phloretin, was optimized using the response surface methodology. The conditions resulting in the maximal yield of phloretin were 14.4 min extraction time, 10% sonication amplitude and 10 g sample per 100 mL 70% (*w*/*w*) ethanol:water solution, while keeping the extraction temperature < 25 °C and retaining a high mixing rate during the extraction process. The highest antioxidant activity was found in the leaves of a local Estonian winter cultivar, ‘Tellissaare’. The phloretin concentration was the highest in the leaves of the winter cultivar ‘Paide taliõun’, at 726 µg/g, and the lowest (292 µg/g) was found in a popular cultivar, ‘Cortland’, originating from the US. Considering the large amounts of leaves available during the summer pruning season in orchards, and the effectiveness of UAE, the local apple tree leaves can be a valuable source of plant polyphenolic compounds to be used to increase the antioxidant activity of functional food.

## Figures and Tables

**Table 1:** Polyphenols extraction from apple leaves and wood.

Material	Apple Cultivar	Solvent	Sample to Solvent Ratio (mg/mL)	Extraction Procedure	Total Phenolic Content *	Phloridzin Content *	Phloretin Content *	Reference
Lyophilized leaf powder	Aldas	70% Ethanol	25	Extraction in US bath at 60 °C for 40 min.	163.35 ± 4.36	106.01 ± 4.23	1.81 ± 0.07	[8]
	Auksis				98.81 ± 1.51	108.90 ± 4.32	1.52 ± 0.06	
	Ligol				107.93 ± 2.94	114.43 ± 4.72	2.40 ± 0.09	
	Lodel				159.86 ± 4.02	109.51 ± 4.62	1.40 ± 0.06	
Lyophilized leaf powder	Szampion	Acidified 30% Methanol (1% HCl)	20	15 min in US bath, 24 h at 4 °C, and 15 min in sonication water bath.	73.33	Dihydrochalcones 36.76		[22]
	Ozarak Gold				115.82	89.85		
Lyophilized leaf powder	Red Fuji	Petroleum Ether	67	3 successive extractions with the different solvents by boiling for 2 h each time.	7.23 ± 0.78	n.a. **	n.a.	[23]
		Ethyl Acetate			25.17 ± 1.52	n.a.	n.a.	
		75% Ethanol			56.74 ± 2.80	66.1	n.a.	
Lyophilized leaf powder	Ozarak Gold	Acidified 80% Methanol (1% acetic acid)	50	15 min in US bath, 24 h at 4 °C, and 15 min in US bath.	160.93	110.15 ± 2.43	n.a.	[12]
Dried leaves at 55 °C	Red Chief	50% Methanol	50	Vortexing for 2 min repeated 2 times after renewing the solvent.	24.10 ± 0.11	21.90 ± 0.02	0.17 ± 1.13	[24]
		70% Methanol			24.48 ± 0.29	18.79 ± 0.08	0.12 ± 0.29	
		50% Ethanol			25.38 ± 0.38	21.07 ± 0.06	0.13 ± 0.07	
		70% Ethanol			30.38 ± 0.50	24.43 ± 0.05	0.15 ± 0.05	
Air-dried leaves powder	Golden	Ethanol	500	Extraction 5 times with ethanol (1 L × 1, 500 mL × 4) at room temperature for 24 h each time.	37.1	52.0	n.a.	[25]
	Royal				34.5	20.3	n.a.	
Apple tree wood dried 22 h at 50 °C, then 4 h at 103 °C	King Jonagold	60% Ethanol	5	Microwave-assisted extraction, 100 °C for 20 min.	Core: 23.8 ± 0.8 Bark: 38.7 ± 1.4 Root: 44.4 ± 2.2	6.89 ± 0.55 20.92 ± 1.09 25.21 ± 1.26	0.195 ± 0.006 0.275 ± 0.006 0.399 ± 0.020	[26]

* Values are expressed as mg/g of dry weight, means ± standard deviation. ** n.a. concentration data not available in the literature.

**Table 2:** Input factors for the Box–Behnken design.

Run	Extraction Time (A, min)		Sonicator Amplitude (B, %)		Sample Weight per 100 mL Solvent (C, g)	
	Real Value	Coded Level	Real Value	Coded Level	Real Value	Coded Level
1	30	1	55	0	10	1
2	17.5	0	55	0	5.5	0
3	30	1	55	0	1	−1
4	5	−1	100	1	5.5	0
5	17.5	0	55	0	5.5	0
6	17.5	0	55	0	5.5	0
7	17.5	0	100	1	10	1
8	30	1	10	−1	5.5	0
9	17.5	0	10	−1	10	1
10	5	−1	55	0	10	1
11	17.5	0	10	−1	1	−1
12	17.5	0	55	0	5.5	0
13	5	−1	10	−1	5.5	0
14	17.5	0	100	1	1	−1
15	30	1	100	1	5.5	0
16	17.5	0	55	0	5.5	0
17	5	−1	55	0	1	−1

**Table 3:** Transition list and MS parameters used for the analysis of phenolic compounds.

				Quantifier *m*/*z*		Qualifier	
Compound	Retention Time (min)	Parent Ion [M+H]+	Parent Ion [M−H]−	CE * V	Production *m*/*z*	CE V	Production *m*/*z*
Chlorogenic acid	6.00	355		−13	166	−41	117
*p*-Coumaric acid	9.00	165		−12	147	−20	119
Caffeic acid	6.65		179	17	135	32	88
Epicatechin	7.77	291		−14	139	−15	123
Phloridzin	15.14	437		−39	107	−12	275
Phloretin	19.65	275		−15	107	−11	169
Rutin	13.65	611		−20	303	−12	465
Quercetin-3-glucoside	13.75	465		−13	303	−55	153
Quercetin-3-galactoside	13.45	465		−13	303	−55	153
Kaempferol-3-glucoside	12.26	449		−53	153	−13	287
Quercetin-3-rhamnoside (quercitrin)	15.34		447	25	301	45	271

* CE: Collision Energy.

**Table 4:** Polyphenol content and antioxidant activity in the extracts from air-dried leaves of different apple cultivars.

Cultivar	Total Polyphenols		Flavonols	DPPH Radical Scavenging Activity	
	mg GA eq./g ^1^	mg ChlA eq./g ^2^	mg Q eq./g ^3^	% ^4^	mg GA eq./g ^1^
Cortland	37.92 ^c^	43.46 ^d^	8.90 ^c^	30.4 ^c^	6.54 ^c^
	±0.38	±0.22	±0.08	±0.5	±0.12
Karksi renett	43.90 ^b^	50.76 ^b^	10.10 ^b^	40.1 ^b^	9.19 ^b^
	±0.83	±0.53	±0.08	±1.1	±0.26
Liivi kuldrenett	37.49 ^c^	39.60 ^e^	9.37 ^d^	30.2 ^c^	6.46 ^c^
	±1.29	±1.26	±0.48	±0.5	±0.18
Paide taliõun	42.04 ^b^	46.20 ^c^	7.47 ^d^	37.9 ^b^	8.35 ^b^
	±0.33	±0.12	±0.09	±0.1	±0.08
Talvenauding	37.00 ^c^	39.60 ^e^	9.37 ^c^	28.9 ^c^	6.06 ^c^
	±1.65	±1.26	±0.48	±1.2	±0.32
Tellissaare	57.74 ^a^	71.06 ^a^	12.23 ^a^	48.4 ^a^	11.42 ^a^
	±0.99	±1.25	±0.05	±0.8	±0.23
Tiina	35.67 ^c^	37.28 ^e^	8.70 ^c^	31.0 ^c^	6.65 ^c^
	±0.52	±0.08	±0.08	±1.1	±0.25

^1^ By FC method (mg GA eq./g: mg gallic acid equivalent/g); ^2^ By UPLC method (mg ChlA eq./g: mg chlorogenic acid equivalent/g of dry weight); ^3^ mg Q eq./g: mg quercetin equivalent/g; ^4^ % of scavenged DPPH radical. All concentrations are expressed per g of the dry weight. All values are means ± standard deviation, *n* = 3; mean values within a column with different letters are significantly different at *p* < 0.05.

**Table 5:** Individual phenolic compounds contents in extracts of air-dried leaves from different apple cultivars.

Phenolic Compounds	Cortland	Karksi Renett	Liivi Kuldrenett	Paide Taliõun	Talvenauding	Tellissaare	Tiina
Chlorogenic acid	227 ± 7 ^cd^	296 ± 14 ^b^	178 ± 2 ^d^	208 ± 3 ^d^	246 ± 7 ^c^	204 ± 2 ^de^	338 ± 11 ^a^
*p*-Coumaric acid	18 ± 1 ^d^	25 ± 1 ^b^	25 ± 1 ^b^	30 ± 1 ^a^	18 ± 1 ^d^	23 ± 1 ^c^	24 ± 1 ^bc^
Caffeicacid	15 ± 1 ^d^	23 ± 1 ^b^	16 ± 1 ^d^	27 ± 1^a^	20 ± 1^c^	17 ± 1 ^cd^	20 ± 1 ^bc^
Phloridzin	17,696 ± 211 ^c^	20,059 ± 173 ^b^	15,491 ± 39 ^d^	18,476 ± 64 ^c^	11,511 ± 406 ^f^	31,654 ± 148 ^a^	12,351 ± 373 ^e^
Phloretin	292 ± 7 ^e^	492 ± 3 ^b^	466 ± 6 ^c^	726 ± 7 ^a^	298 ± 5 ^e^	505 ± 4 ^b^	360 ± 2 ^d^
Quercetin- 3-glucoside	940 ± 11 ^c^	1554 ± 8 ^b^	637 ± 16 ^e^	447 ± 20 ^g^	515 ± 19 ^f^	1915 ± 15 ^a^	849 ± 12 ^d^
Quercetin- 3-galactoside	220 ± 11 ^d^	896 ± 30 ^a^	553 ± 9 ^b^	360 ± 8 ^c^	409 ± 13 ^c^	859 ± 16 ^a^	540 ± 14 ^b^
Quercetin-3-rhamnoside	2248 ± 32 ^cd^	1994 ± 23 ^e^	3530 ± 44 ^b^	1988 ± 1 ^e^	2133 ± 70 ^d^	2289 ± 31 ^c^	4290 ± 11 ^a^
Rutin	198 ± 3 ^c^	426 ± 4 ^a^	86 ± 1 ^e^	48 ± 1 ^f^	143 ± 4 ^d^	313 ± 2 ^b^	21 ± 1 ^g^
Kaempferol- 3-glucoside	979 ± 10 ^d^	943 ± 15 ^e^	1377 ± 6 ^b^	809 ± 8 ^f^	866 ± 38 ^f^	1162 ± 21 ^c^	1680 ± 34 ^a^

All concentrations are expressed as µg per g of the dry weight. All values are means ± standard deviation, *n* = 3; mean values within a row with different letters are significantly different at *p* < 0.05.

## Data Availability

We did not include such data in this study.
